# Biomechanical analysis of marathon shoes applied to NESTFIT technology

**DOI:** 10.1186/1757-1146-7-S1-A122

**Published:** 2014-04-08

**Authors:** Seung-Bum Park, Kyung-Deuk Lee, Dae-Woong Kim, Jung-Hyeon Yoo, Kyung-Hun Kim, Jin-Hoon Kim

**Affiliations:** 1 Footwear Biomechanics Team, Footwear Industrial Promotion Center, Busan, Korea; 2 Design Center, Treksta INC, Busan, Korea

## 

The purpose of this study was to analyze foot pressure distribution of marathon shoes to which NESTFIT Technology was applied. As for marathon, shoes play a vital role in shortening records. However, they also might become a main factor of injury during long-distance running. This study will examine foot pressure distribution effects of marathon shoes during long-distance running, which have been developed by measuring Korean shoe lasts.

The methods of this study can be explained as below. Firstly, ten healthy males were picked as subjects to participate in this study. 10 healthy male subjects with an average age of 22.3 years (SD=0.5), weight of 71.5 kg (SD=6.0) and height of 173.1 cm (SD=4.3) were recruited for this study. Secondly, the one equipment used for the study consist of a foot pressure device from Pedar-X, Germany and a treadmill from Pulse fitness, UK. Thirdly, the testing procedures involve each subject to test three different shoes by having running trials on a treadmill at a constant speed of 12.0km/hour.

**Figure 1:**
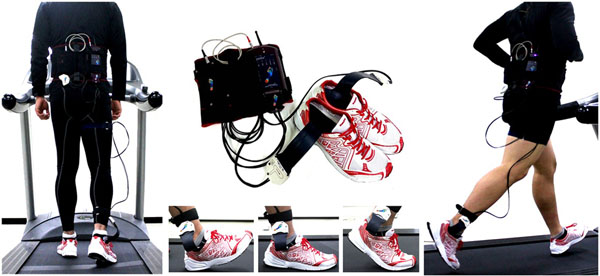
Marathon Shoes (NESTFIT Technology)

The pressure distribution data (contact area, maximum force, maximum peak pressure, maximum mean pressure) was collected by using a pressure device at a sampling rate of 100Hz. The statistical analysis was carried out by using the MINITAB R15 package, specifically One-way ANOVA (α=.05). Type A shoe has the lowest peak pressure at total mask. Generally, the Type A shoe had overall lower values for the maximum force and maximum mean pressure variables compared to Type B, C shoe conditions.

In comparison with the control group: 1)The contact areas of foot (total) increased 0.87% than Type B, midfoot increased 5.17% than Type B and 0.59% than Type C. 2) The maximum force of foot (total) decreased 4.39% than Type B and 2.72% than Type C, rearfoot decreased 5.42% than Type B and 2.72% than Type C. 3)The maximum peak pressure of midfoot decreased 6.64% than Type B and 11.46% than Type C, rearfoot 4.36% than Type B and 10.66% than Type C. 4)The maximum mean pressure of foot (Total) decreased 1.74% than Type B and 1.79% than Type C, rearfoot decreased 36% than Type B and 10.66% than Type C. 

**Table 1:** Result of Foot Pressure (Marathon Shoes: Type A)

Mask	Contact area (cm^2^)				Maximum force (N)			
	
	A	B	C	*p*-value	A	B	C	*p*-value
Total	156.62±23.25	155.27±17.25	156.92±12.07	0.98	1,309.43±252.33	1,369.60±183.88	1,345.98±121.75	0.78
Forefoot	60.42±9.02	60.99±7.12	61.03±4.66	0.98	746.65±170.96	792.76±121.39	728.42±101.16	0.55
Midfoot	54.40±9.13	51.73±7.31	54.08±5.67	0.69	436.86±114.82	398.41±105.88	411.33±95.55	0.71
Rearfoot	41.04±5.32	41.80±3.86	41.07±3.22	0.90	501.65±109.75	530.39±96.21	537.09±79.32	0.68

**Mask**	**Maximum peak pressure (kPa)**				**Maximum mean pressure (kPa)**			
	
	**A**	**B**	**C**	***p*-value**	**A**	**B**	**C**	***p*-value**

Total	298.03±85.12	256.10±256.10	266.04±266.04	0.31	94.27±18.45	95.94±11.20	95.99±10.74	0.95
Forefoot	289.14±85.26	248.23±41.31	252.43±47.66	0.28	119.79±28.58	127.51±20.42	118.60±16.84	0.63
Midfoot	147.34±43.97	157.82±49.05	166.41±43.38	0.65	77.87±21.35	75.85±19.14	76.45±17.28	0.97
Rearfoot	185.35±68.01	193.80±53.62	207.45±52.65	0.70	120.43±23.54	126.65±22.14	130.28±18.08	0.59

* *p*<.05

**Figure 2:**
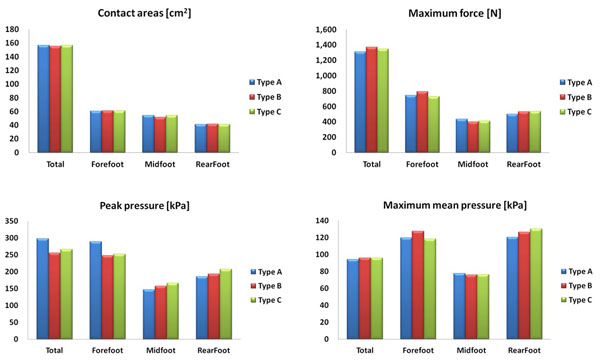
Result of Foot Pressure (Marathon Shoes: Type A)

As a result of analysis, it has been found that Type A has the lower maximum force (total) and maximum mean pressure (total) than Type B or C. Also, it has been found that Type A has the lower maximum force (rearfoot) and foot pressure (rearfoot) than Type B and Type C. In addition, it has been proved that the maximum force and maximum mean pressure of Type A is lower than any other control groups so that it provides pressure distribution effects during long-distance running.
